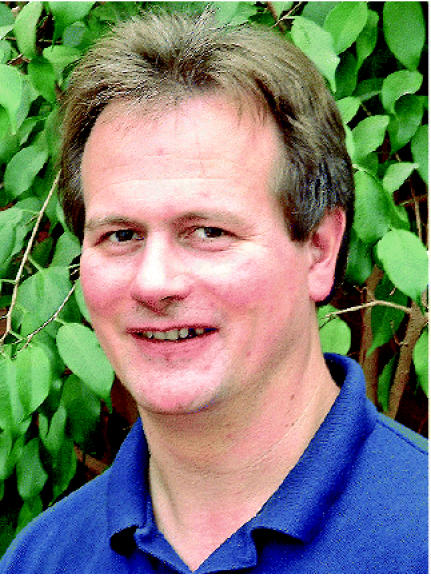# Inflammatory Genomics

**DOI:** 10.1289/ehp.113-a794

**Published:** 2005-12

**Authors:** Timothy W. Gant

**Affiliations:** Medical Research Council Toxicology Unit, University of Leicester, Leicester, United Kingdom, E-mail: twg1@le.ac.uk

As a University of London undergraduate beginning a module on pathology, I remember Professor Frank Fairweather opening his lecture by pointing to a large boil on his forehead as an example of acute inflammation. He then proceeded to describe the gross pathological characteristics of acute inflammation: weal, brief blood vessel constriction, followed by blood vessel dilation and associated redness. Such was my introduction to the most common consequence of tissue damage—and contributor to disease pathogenesis—inflammation.

Inflammation is mediated by chemical activators, collectively known as chemokines, secreted in the area of the tissue damage. Chemotactant proteins are expressed on the endothelial cell of the dilated blood vessels that serve as recruitment factors for lymphocytes. Blood vessel dilation causes a decrease in local blood flow, and activated neutrophils, attracted by the chemokines, attach to the chemotactant proteins, squeeze themselves through the endothelial cell walls of the locally dilated blood vessels, and follow the scent of the chemokines to the site of damage (for additional information, see Schmidt 2005).

Toxicogenomics has led to an additional description of inflammation based on the differential expression of genes associated with the inflammatory process. One of the first toxicogenomics reports published was that of the differential expression of genes in response to lipopolysaccharide-induced inflammation (Heller et al. 1997). Several reports now link the expression of certain genes, in particular the attachment genes *Vcam1* and *Icam1*, to, for example, inflammation in the liver (Davies et al. 2005; Gant et al. 2003; Huang et al. 2004; Jiang et al. 2004). To date, a quantitative fingerprint of gene expression associated with inflammation has not been defined. In the GeneOntology (GO) database (GO 2005), genes associated with, but not necessarily quantitative for, inflammation are identified in biological processes as “inflammatory response.” Under inflammatory response in the GO, there are 371 genes listed for *Homo sapiens*. Tumor necrosis factor-α(*TNF-*α) is included among these 362 genes, but interleukin 6 (*IL-6*) is not, although IL-6 is used as a plasma biomarker of inflammation (Curran et al. 2005). Similarly, a recent study in the liver has associated three genes *PGS6* (pregnancy-specific β-1-glycoprotein), *GSTM4/M2* (glutathione *S*-transferase mu 4 and mu 2), and *OAT* (ornithine ketoacid aminotransferase) with inflammation in human liver (Younossi et al. 2005); these genes, like *IL-6*, are not categorized as inflammation genes in the GO. Thus, not all genes associated with inflammation are defined as such in GO, and none are quantitatively associated. Therefore, to provide a repository of data for making future associations, we need a maintained sub-database of differential gene expressions that are quantitatively associated with measured pathological responses. Such quantitative association of gene expression with altered pathology, known as “phenotypic anchoring” (Moggs 2005; Moggs et al. 2004; Paules 2003; Waters and Fostel 2004), includes measurement of both gene expression and degree of pathological change. Few data sets in Gene Expression Omnibus (GEO 2005) or ArrayExpress (European Bioinformatics Institute 2005) contain a histopathological quantitation of inflammation of sufficient quality to allow retrospective phenotypic anchoring of differential gene expression at the present time. More data sets need to include an actual measure of pathological change. In particular, toxicogenomic data should be collected before and during the onset of measured pathological change.

However, before embarking on the development of a phenotypically anchored database of signature gene expression, we must ask the following question: Does toxicogenomics have any advantage over histopathology in the assessment and characterization of pathological change? For inflammation, as for other pathologies, the answer to this question depends on whether toxicogenomics can *a*) detect inflammation before it becomes histopathologically observable, *b*) provide a more quantitative assessment of its severity, and *c*) distinguish between the acute and chronic forms and other pathologies. If we are referring to the most informative genes, the answer to these questions is probably “yes,” but more data is necessary to derive conclusive answers. Thus, the generation of more gene expression data is necessary in targeted pathologies such as inflammation, and a phenotypically anchored database should be targeted to specific common pathologies in the first instance so critical data masses of gene expression data can be collected.

In the early development of microarrays and their application in toxicology, some predictions were made that histopathologists would become an endangered species, made redundant by the new technology. This has not happened, and even the reverse could be argued to have occurred; toxicogenomics has proven so challenging for interpretation that there has been a retreat into the “gold standard” methods of analysis (Albertini 2005). Toxicogenomics has the potential to inform and append histopathological assessment, injecting a degree of instrumental precision into the analysis and assisting in the differentiation of difficult-to-discern lesions (Gant 2002, 2003; Lakhani and Ashworth 2001). Although there is still much work to be done, toxicogenomics will gradually gain a central role in the toxicologists’ armory—as long as expectations are reasonable, quality is good, interpretation is expert, and conclusions are balanced. Genomics has much to offer in pathological assessment, but its application should be collaborative, not inflammatory.

## Figures and Tables

**Figure:**